# Impaired Integrated Stress Response and Mitochondrial Integrity Modulate Genotoxic Stress Impact and Lower the Threshold for Immune Signalling

**DOI:** 10.3390/ijms24065891

**Published:** 2023-03-20

**Authors:** Mihaela Temelie, Rubab Talpur, Marta Dominguez-Prieto, Ayanda Dantas Silva, Constantin Cenusa, Liviu Craciun, Diana Iulia Savu, Nicoleta Moisoi

**Affiliations:** 1 Department of Life and Environmental Physics, Horia Hulubei National Institute for R&D in Physics and Nuclear Engineering, Reactorului 30, P.O. Box MG-6, 077125 Magurele, Romania; 2 Leicester School of Pharmacy, Leicester Institute for Pharmaceutical Innovation, Faculty of Health Sciences, De Montfort University, The Gateway, Hawthorn Building 1.03, Leicester LE1 9BH, UK; 3 Radioisotopes and Radiation Metrology Department, Horia Hulubei National Institute for R&D in Physics and Nuclear Engineering, P.O. Box MG-6, 077125 Magurele, Romania; 4 Applied Nuclear Physics Department, Horia Hulubei National Institute for R&D in Physics and Nuclear Engineering, Reactorului 30, P.O. Box MG-6, 077125 Magurele, Romania

**Keywords:** DNA damage, mitochondria function, integrated stress response, radioinduced signalling pathways

## Abstract

Mitochondria–nucleus communication during stress dictates cellular fate with consequences on the etiopathology of multiple age-related diseases. Impaired mitochondrial quality control through loss of function of the mitochondrial protease HtrA2 associates with accumulation of damaged mitochondria and triggers the integrated stress response, implicating the transcription factor CHOP. Here we have employed a combined model of impaired mitochondria quality control, namely HtrA2 loss of function, and/or integrated stress response, namely CHOP loss of function, and genotoxicity to address the distinctive roles of these cellular components in modulating intracellular and intercellular responses. The genotoxic agents employed were cancer therapeutic agents such as irradiation with X-ray and protons or treatment with the radiomimetic bleomycin. The irradiation had an enhanced effect in inducing DNA damage in cells with CHOP loss of function, while the bleomycin treatment induced more DNA damage in all the transgenic cells as compared to the control. The genetic modifications impaired the transmission of DNA damage signalling intercellularly. Furthermore, we have dissected the signalling pathways modulated by irradiation in selected genotypes with RNA sequencing analysis. We identified that loss of HtrA2 and CHOP function, respectively, lowers the threshold where irradiation may induce the activation of innate immune responses via cGAS-STING; this may have a significant impact on decisions for combined therapeutic approaches for various diseases.

## 1. Introduction

Mitochondria dysfunction and DNA damage accumulation are central to ageing and age-related diseases including neurodegeneration, cancer, and metabolic disorders. Alongside other cellular perturbations including accumulation of oxidative damage, impaired autophagy, proteostasis, dysregulated metabolic pathways, and bioenergetics, they contribute to abnormal cellular homeostasis and imbalance in cell death/cell survival mechanisms as well as enhanced inflammation and senescence [1]. Mitochondria–nucleus communication and its integration within the maintenance of cellular homeostasis constitute key determinants of cellular fate and organism health in the context of cellular stress, homeostasis perturbation, and disease [2].

The cellular response to genotoxic stress involves a plethora of signalling mechanisms that may have pro-survival or pro-death consequences. A multitude of these signalling pathways controlling cell death and survival have been effectively investigated in tumorigenesis [3] and have been proven more recently to be linked with the etiopathogenesis of neurodegenerative diseases [2]. The final cellular outcome is dictated by the DNA damage response (DDR) pathways and DNA repair mechanisms triggered to remove nuclear DNA lesions [4]. Insult on other cellular compartments triggers stress response mechanisms including unfolded protein response (UPR) mediated by organelles such as the endoplasmic reticulum (ER) and mitochondria, cytosolic heat shock response (HSR) [5,6,7], and integrated stress response (ISR) [8]. These stress pathways act either independently or in an overlapping manner, influencing the cell survival/death rate. Besides the intracellular roles of stress signalling mechanisms, there is now significant evidence for intercellular signalling of stress. The intercellular stress consequences are effectively copying the direct effects, whether these consist of damage- or stress-signalling initiation. Thus, the cells undergoing DNA damage communicate with the nontreated cells by the so-called bystander effect [9] and by unfolded protein stress response acting non-autonomously in distant cells [2].

C/EBP homologous protein (CHOP), also known as DNA damage inducible transcript 3 and as DNA damage-inducible protein 153 (GADD153), is induced by multiple stressors including genotoxic agents/DNA damage, growth arrest, nutrient deprivation, and hypoxia. CHOP acts as a key regulator of ER stress, integrated stress response, and mitochondrial stress. The response to stress can lead to changes in gene expression patterns alongside a general shutdown and reprogramming of protein synthesis. In these conditions, ribosomes selectively recruit mRNA, whose protein products are necessary for the buildup of the stress response. Translational reprogramming to sustain the response to stress may result in increased lifespan, and its deregulation is associated with disease. Through initiation of transcriptional stress responses, CHOP controls numerous genes involved in varied cellular processes including inflammation, differentiation, autophagy, and apoptosis [10].

Regulation of CHOP depends on the stress pathway that is triggered by the stress challenge. In the case of integrated stress signalling, this typically involves the upregulation of CHOP downstream of activating transcription factor 4 (ATF4). In the case of ER stress, its transcription is regulated by ER stress response elements and the C/EBP-ATF response element. In amino acid starvation, the regulation takes place through amino acid response elements. Recent work in cardiac cells has detailed an interplay between CHOP, ATF4, and C/EBP following mitochondrial dysfunction in which CHOP works to attenuate an overactive ISR by reducing ATF4 at transcriptional level in a feedback loop [11].

Markers of ISR activation alongside markers of mitochondrial stress signalling have been identified in age-related neurodegenerative diseases including in Parkinson’s disease (PD). Thus, the loss of the intermembrane space mitochondrial protease HtrA2 leads to accumulation of unfolded proteins in the mitochondria, impaired mitochondrial respiration, accumulation of reactive oxygen species (ROS), and a neurodegenerative disorder with Parkinsonian phenotypes in Htra2 knockout (KO) mice [12]. These are associated with the specific activation of the ISR specifically in the brain, including upregulation of CHOP, without enhancing markers of classical ER stress (e.g., binding immunoglobulin protein 78 (BiP/Grp78)) or mtUPR (e.g., heat shock protein 60 (Hsp60), caseinolytic peptidase (CLPP)). HtrA2 loss of function has been linked to accelerated brain ageing and neurodegeneration [13], and we have been able to confirm the activation of ISR in the HtrA2 KO brains and in idiopathic PD brain samples [14]. These findings have raised the question of what is the consequence of the ISR activation to the neurodegenerative process. To this end, we have shown that removing CHOP in the HtrA2 KO protects against the neurodegenerative process, indicating that prolonged activation of ISR and CHOP upregulation contributes to cell death.

Besides their involvement in neurodegenerative diseases, both HtrA2 and CHOP as an ISR component were found to be involved in cancer. The HtrA2 role in neurodegeneration is related to its loss of function in the mitochondrial intermembrane space. However, HtrA2 can transfer to the cytoplasm through loss of its mitochondrial targeting sequence. Here HtrA2 targets inhibitor apoptosis proteins having a major role in regulating apoptosis. The expression of HtrA2 changes in oncogenesis and is upregulated or downregulated in tumor cells depending on the type of cancer [15]. ISR has a higher basal level in tumour cells than in normal cells due to the higher rate of mitosis; its roles in tumour etiopathogenesis and progression appear to vary depending on tumour type. Thus, therapeutic strategies are being designed to modulate ISR in tumour treatments either in enhancing or reducing its activation depending on the context. The upregulation of CHOP appears to shift the balance towards apoptosis and autophagy through ER stress in tumour cells, contributing to cell death [16]. CHOP loss of function has also been shown to protect cells from ER stress induced by tunicamycin by decreasing ER client protein load and changing redox conditions within the organelle. While deletion of CHOP did not abrogate the upregulation of ER stress genes completely in previous studies, it worked to delay this response and had a protective effect overall on cellular viability, thus leading to the conclusion that CHOP sensitises cells to ER stress-mediated death [17].

Here we have investigated the effect of genotoxic stress induced by irradiation of different qualities (photons and protons) and chemotherapeutic bleomycin (BLM) in the context of impaired CHOP signalling and mitochondrial quality control dysfunction through HtrA2 loss of function. The loss of integrity of the ISR and mitochondrial function affects both the response to the direct effect of genotoxic treatments and the transmission of bystander effects. Thus, we show for the first time that CHOP and HtrA2 play a role in the transmission of intercellular signalling of genotoxic stress. Moreover, we show that ISR loss of function as well as mitochondrial quality control impairment accelerate stress response mechanisms initiated by DNA damage, most significantly the acceleration of cGAS-STING innate immune pathways.

## 2. Results and Discussion

### 2.1. Mitochondrial Homeostasis Is Influenced by Mitochondrial Quality Control and ISR Integrity

**Figure 1:**
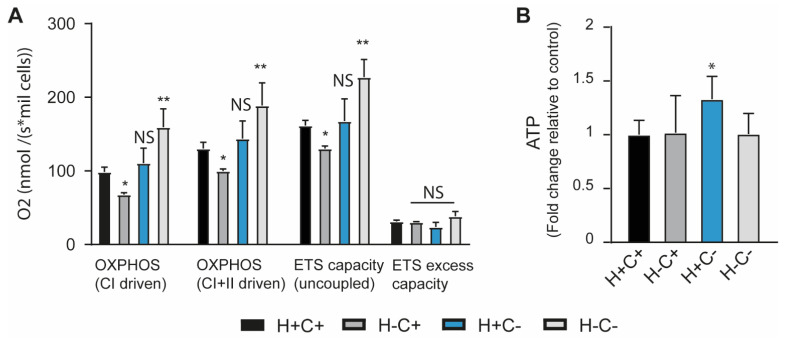
(**A**) Analysis of the respiratory activity shows that this parameter is reduced in the cells with HtrA2 loss of function and increased for combined CHOP/HtrA2 loss of function. The parameters presented are Complex I and Complex I + II–linked respiration in the presence of ADP, ETS capacity, i.e., maximum respiration in uncoupled mitochondria as well as the calculated mitochondrial excess capacity. Each data point represents the mean +/− SD of two independent experiments. The statistical significance was determined with the One-way ANOVA with multiple comparisons and with the Student *t*-test. The statistical significance versus the H+C+ is indicated for each parameter. (**B**) The basal ATP level as measure of mitochondrial function is enhanced in the cells with CHOP loss of function. Each data point represents the mean ± SD of at least three independent experiments. Statistical analysis was performed with One-way ANOVA with multiple comparisons versus H+C+, and statistical significance is indicated in the figure (* *p* < 0.05, ** *p* < 0.01 and NS indicates *p* > 0.05).

**Figure 2:**
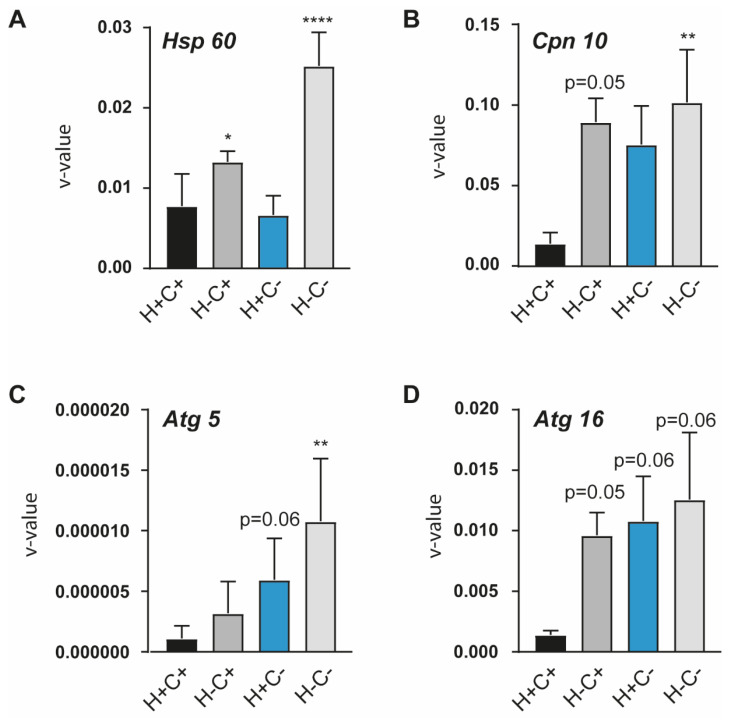
HtrA2 and CHOP loss of function modify the transcriptional level of mitochondrial stress chaperones (Panel (**A**). *Hsp60*, Panel (**B**). *Hsp10*) and mitophagy related atg-s (Panel (**C**). *Atg5*, Panel (**D**). *Atg16*). The data are presented as mean ± SD from three experiments. The statistical significance was analysed with One-way ANOVA with multiple comparison’s and the difference versus the H+C+ genotype is indicated in the figure (* *p* < 0.05, ** *p* < 0.01, **** *p* < 0.0001).

Given the link between mitochondrial physiology and quality control signalling mechanisms, we have analysed mitochondrial respirometry, the baseline ATP level (Figure 1A,B), and the level of key mtUPR and mitophagy transcription factors (Figure 2) in the 4 cell lines in untreated conditions.

To evaluate mitochondrial physiology parameters, we have measured OXPHOS Complex I- and OXPHOS Complex I- and II-driven respiratory states, maximum electron transfer system (ETS) capacity (Figure 1A). All these parameters have indicated a decrease in respiratory activity in the HtrA2 loss-of-function cells, which we have previously seen in HtrA2 KO brain mitochondria [14]. Previous analysis of the effect of HtrA2 loss of function on mitochondrial physiology indicated that the enzymatic activities of the respiratory complexes are not changed. However, the levels of citrate synthase indicated a reduction in the mitochondrial mass associated with morphological changes of the mitochondria, indicative of the accumulation of damaged organelles [12]. We have observed no significant differences in the respiratory parameters for the CHOP loss of function and an increase in respiration in the double KO cells. Thus, it is likely that the double mutation leads to compensatory mechanisms that affect mitochondrial dysfunction events induced by HtrA2 loss of function alone. Interestingly, the ETS excess capacity, reflecting the ability of mitochondria to drive processes other than oxidative phosphorylation without competing with ATP production, is not different between the tested genotypes. Thus, the loss of function of HtrA2 and CHOP impacts primarily the ATP synthesis metabolic pathways. The basal ATP level (Figure 1B), however, appears to be higher in the H+C- genotype, suggesting that for each genotype modification there are distinct compensatory mechanisms to balance the overall bioenergetic homeostasis.

Mitochondrial quality control is largely controlled by two factors: molecular quality control, in which chaperones and proteases are upregulated to resolve the accumulation of unfolded proteins in the mitochondria, and organellar quality control (mitophagy), in which damaged mitochondria are removed through lysosomal degradation [2]. We have employed *Hsp60* and *Hsp10* as markers of unfolded protein stress and have identified that these are upregulated by HtrA2 loss of function both in the presence and absence of CHOP, but they are not modified by the loss of CHOP alone (Figure 2A,B). This supports the role of HtrA2 in clearing unfolded proteins in the mitochondrial intermembrane space which we have previously identified [14], and indicates that loss of ISR signalling may not enhance further the accumulation of unfolded proteins in the mitochondria. We have also verified the upregulation of CHOP induced by HtrA2 loss of function, which we have previously demonstrated (Supplementary Figure S1) [14]. As markers of organellar quality control we have employed autophagy gene 5 and 16 (*Atg5* and *Atg16*), which are involved in the elongation of the autophagosome, thus playing a key role in mitophagy. These markers appear to be increased in all the transgenic lines as compared to the control, indicating potentially distinct routes of activation for mitophagy in the case of mitochondrial dysfunction through loss of HtrA2 and in the case of loss of ISR integrity.

Cells support pro-survival mechanisms following genotoxic damage through irradiation by activating mechanisms which protect/restore mitochondrial function through activation of autophagy and enhanced mitochondrial biogenesis. In addition, multiple signalling pathways are activated to respond to stress. Thus, irradiated cells present increased activation of the integrated stress response consisting of ATF4 and CHOP upregulation. Additional markers of mitochondrial stress, namely accumulation of mitochondrial chaperones, HSP60, HSP10, and the transcription factor ATF5 have also been observed [18].

### 2.2. Sensitivity to Genotoxic Stressors

Given that both mitochondrial dysfunction and DNA damage are hallmarks of ageing and age-related diseases, we employed a combined model of impaired mitochondrial stress signalling and DNA damage for further analysis of the mitochondria–nucleus communication of stress signals.

**Figure 3:**
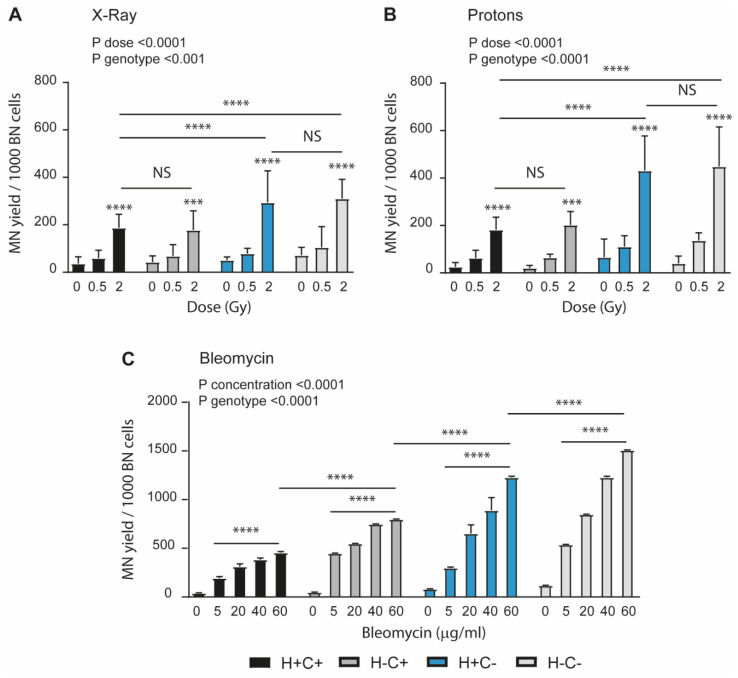
Cellular sensitivity to DNA damage is dependent on the genotoxic agent and genotype. The cells were exposed to X-Ray (Panel (**A**)) and Proton (Panel (**B**)) irradiation or to bleomycin (Panel (**C**)) treatment as indicated. The cells were analysed at 24 h for the direct effects of the genotoxic treatment. The results indicate that CHOP loss of function sensitises the cells to accumulation of DNA damage for the irradiation challenge, while the bleomycin effect is higher in the cells with all mutations. The data are presented as mean ± SD (3–7 independent experiments for each genotype). The statistical analysis was performed with Two-way ANOVA with Tuckey multiple comparisons. The statistical significance versus untreated control is indicated for each genotype alongside comparisons between genotypes at the maximum dose (*** *p* < 0.001, **** *p* < 0.0001 and NS indicates *p* > 0.05).

Direct irradiated treatment with either X-rays (Figure 3A) or protons (Figure 3B) induced a dose-dependent induction of micronuclei (MN) in all the cell lines. However, proton irradiation generated a higher accumulation of MN in MEFs with CHOP loss of function as compared to the X-ray irradiation. The accumulation of MN did not seem to be affected by the HtrA2 loss of function for the X-ray irradiation and the proton irradiation. We have also compared with two-way Anova the induction of MN by X-ray and protons at 2 Gy. The analysis indicates that proton irradiation has a statistically different effect (higher) as compared to X-ray irradiation. The radiomimetic chemical bleomycin induced a much higher effect than irradiation in all the cells with mutations (Figure 3C). Mitochondrial function modulated by HtrA2 and ISR appears to have a role in the different pathways controlling the nuclear DNA stability. In a previous study, our group found evidence of increase in DNA damage due to mitochondrial PINK1 loss of function [19]. Thus, disruption of mitochondrial homeostasis at various levels appears to have essentially the same end result of enhancing genotoxic damage.

**Figure 4:**
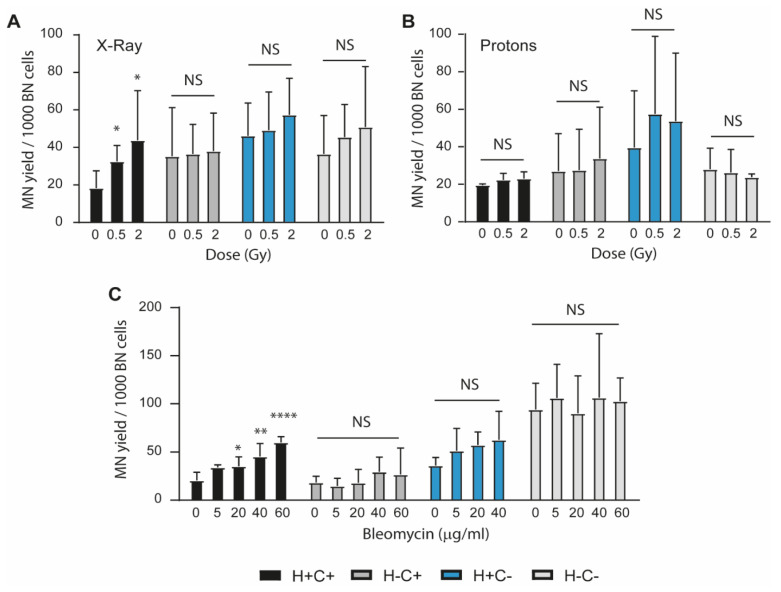
Intercellular bystander signalling of DNA damage requires mitochondrial function and ISR integrity. Accumulation of bystander DNA damage accumulation was evaluated through a media transfer experimental approach after induction of genotoxic stress with X-Ray (Panel (**A**)) and Proton (Panel (**B**)) irradiation or with bleomycin (Panel (**C**)). H+C+ cells are able to transmit bystander effects for X-Ray and bleomycin treatments, while cells with mitochondrial and ISR loss-of- function mutations do not transmit bystander signalling. The cells are not able to transmit bystander stress for any genotype following proton irradiation. The data are presented as mean ± SD (3–7 independent experiments for each genotype). The statistical analysis was performed with two-way ANOVA with Dunnett’s multiple comparisons. The statistical significance versus untreated control is indicated for each genotype (* *p* < 0.05, ** *p* < 0.01, **** *p* < 0.0001 and NS indicates *p* > 0.05).

Bystander DNA damage assessed as MN formation was investigated in bystander MEFs cells (H+C+, H+C-, H-C+, H-C-), which received conditioned medium H+C+ irradiated with X-rays or protons or treated with bleomycin (Figure 4A–C). This study was performed to evaluate the implication of mitochondria fitness on intercellular communication following genotoxic stress. The results showed that in case of X-ray irradiation, the number of MN increased significantly only in bystander H+C+ cells receiving conditioned medium from H+C+ cells, while no modification was observed in any of the mutant cells (Figure 4A). A similar pattern is followed in the case of BLM treatment, when it can be observed that bystander signals are received only by bystander H+C+ cells, not by any bystander mutant cells (Figure 4C). The conditioned medium of proton-irradiated H+C+ cells did not transmit bystander stress to any genotype (Figure 4B). Taken together, this outcome implied that cells with mitochondrial and ISR loss-of-function cells do not receive bystander signals. The occurrence of DNA damage that is not a consequence of the direct effect of irradiation was put into evidence around three decades ago [20]. Intercellular signalling mediated by factors released from irradiated cells is considered a possible mechanism that could trigger the response in neighbouring cells, but the nature of these factors is still unclear. Mitochondrial function appeared to be correlated with triggering the bystander effect. Moreover, the crosstalk between mitochondria and ER in response to IR was proved to be linked to the triggering of bystander response. The activation of mitochondria in human lung fibroblast cells after IR hitting only the nucleus with a proton microbeam was reported. The activation of mitochondria was inhibited with rotenone, and consequently the bystander MN were also found reduced. In addition, following the IR-hitting cytoplasm, the ER mass increased; further, when the cells were treated with rotenone together with depressing the ER chaperone BiP, the MN level increased [21]. We have also shown that mitochondrial dysfunction through PINK1 loss of function (in directly and bystander genotoxic treated cells) impairs both intracellular and intercellular bystander signalling [19]. Thus, similarly with the induction of direct genotoxic damage, disruption of mitochondrial homeostasis at various levels appears to have essentially the same end result of impairing bystander signalling.

### 2.3. Signalling Mechanisms Revealed by RNA Sequencing

We have performed RNA sequencing analysis for selected conditions for the HtrA2 and CHOP loss of function (Supplementary Table S1). iDEP differential expression analysis identified upregulated and downregulated genes (Supplementary Table S2 and Figure S2) which we have further subjected to pathway enrichment analysis, revealing significant differences in signalling mechanisms triggered by irradiation in the control versus the transgenic cells (Supplementary Table S3).

**Figure 5:**
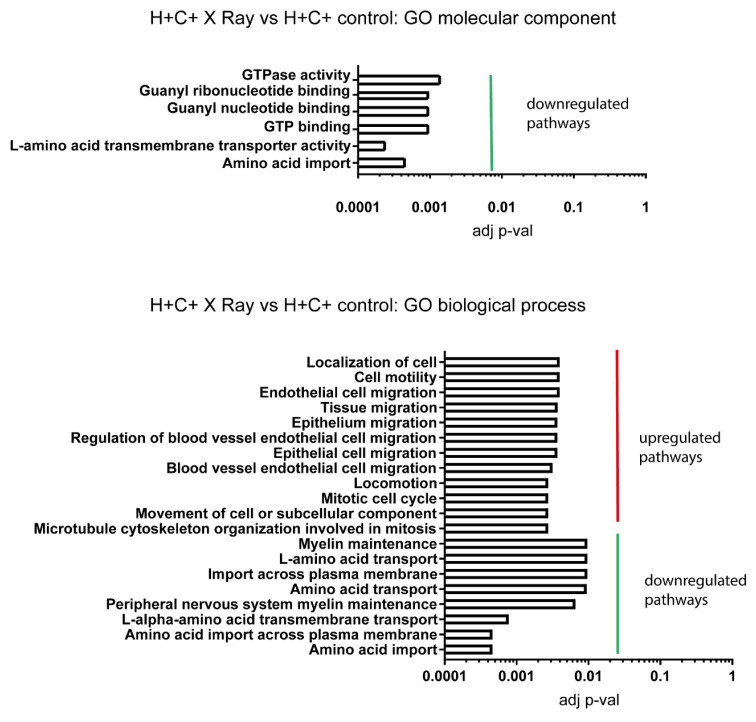
Enrichment analysis for X-ray irradiated H+C+ versus untreated control. GO molecular component and GO biological process have provided significantly enriched pathways.

**Figure 6:**
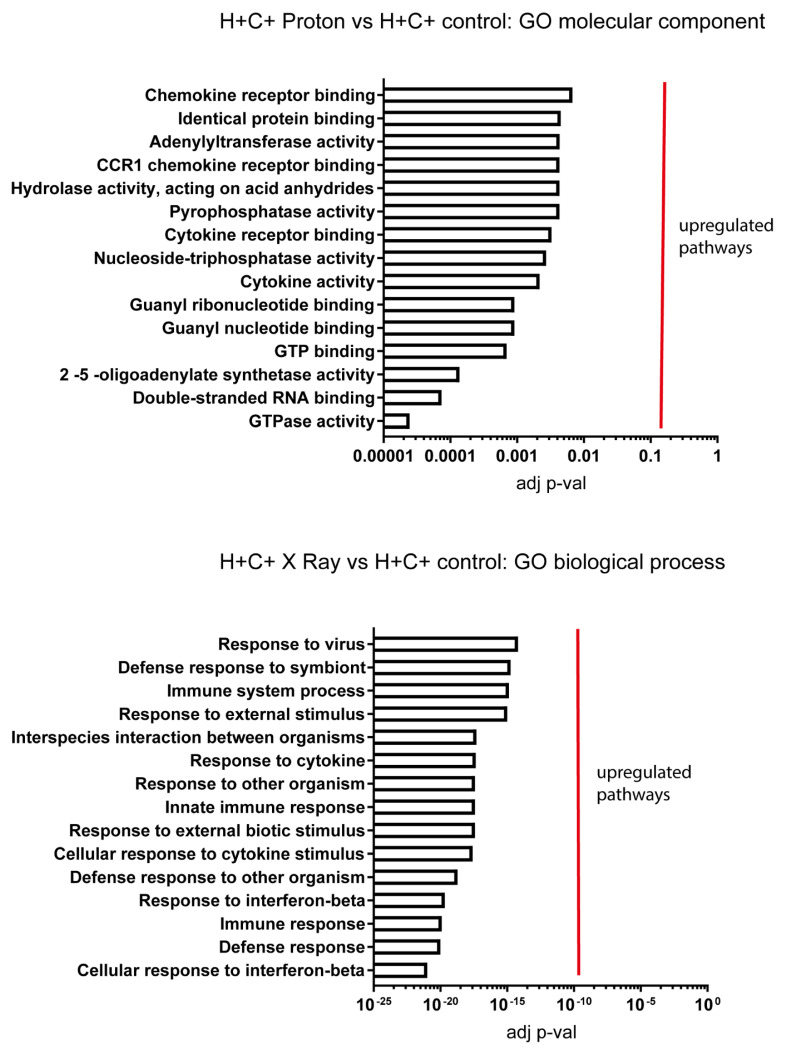
Enrichment analysis for proton-irradiated H+C+ versus untreated control. GO molecular component and GO biological process have provided significantly enriched pathways.

Thus, X-ray irradiation in H+C+ cells downregulates GTP-related enzymes, which may result in impaired G-protein coupled signalling and downregulate amino acid transporters (Figure 5). Both processes appear at the level of the plasma membrane. In addition, this type of stress upregulates genes involved in cellular motility and mitosis involving cytoskeleton rearrangements. The effect of proton irradiation in H+C+ control cells reflects the enhanced effectiveness characteristic of this type of irradiation as compared to the X-ray irradiation. The increased damage process at the nuclear DNA level is indicated by the upregulation of pathways involved in DNA repair mechanisms as well as in the activation of an innate immune response, which has been shown to be triggered by DNA damage that affects both nuclear and mitochondrial DNA (Figure 6). This aspect of cellular signalling is highly significant in demonstrating the distinct mechanisms triggered by the different physical characteristics at the same dose.

**Figure 7:**
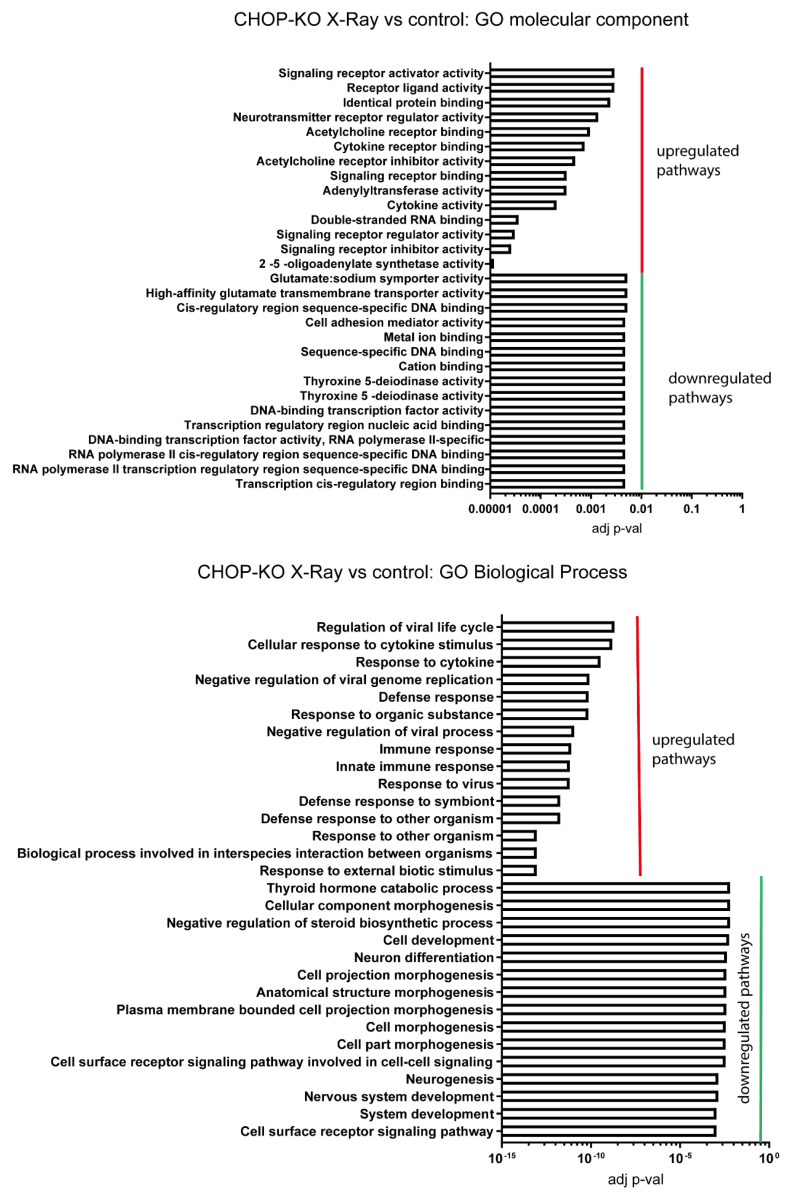
Enrichment analysis for X-ray irradiated H+C- versus untreated control. GO molecular component and GO biological process have provided significantly enriched pathways.

In CHOP KO cells (Figure 7), the impairment in the ISR signalling induces upregulation of pathways related to receptor activations and innate immunity, suggesting that the ISR loss of function lowers the threshold at which DNA damage leads to an immune response. Interestingly, DNA and RNA transcripts appear to be mainly downregulated, indicating a halt in protein transcription and translation and suggesting that the effect of ISR activation is not abolished but only reduced; this has been previously demonstrated in CHOP KO cells under ER stress triggered with Tunicamycin treatment [17]. Moreover, pathways related to changes in cellular morphology appear to be downregulated, indicating a state of ‘slowdown’ in a range of cellular processes.

**Figure 8:**
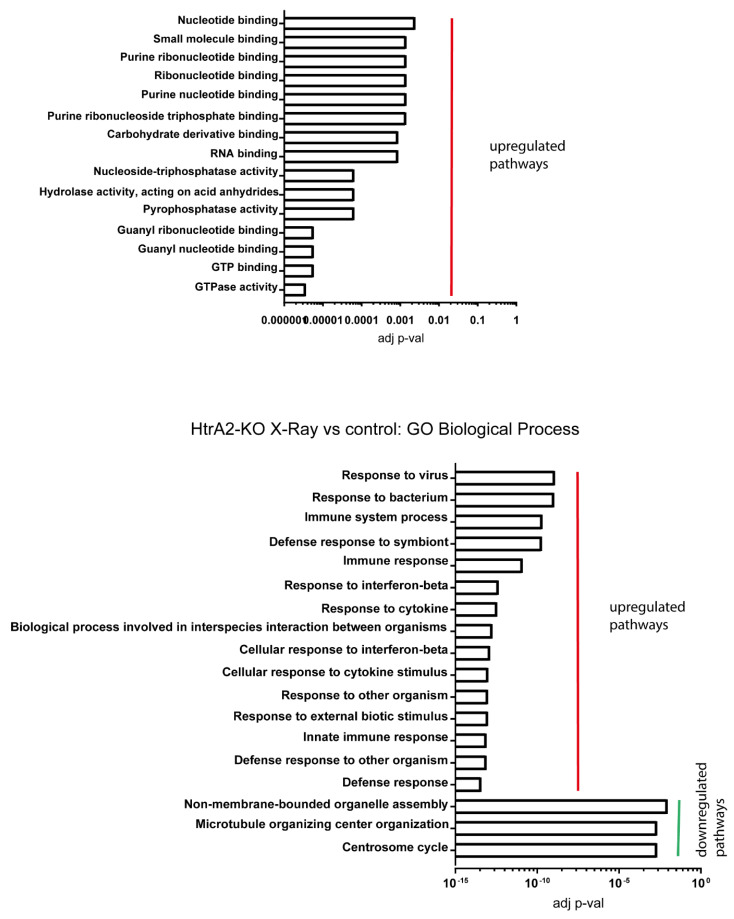
Enrichment analysis X-ray irradiated H-C+ for versus untreated control. GO molecular component and GO biological process have provided significantly enriched pathways.

The X-ray irradiation of HtrA2 KO cells (Figure 8) indicates that the signalling of DNA and RNA binding mechanisms is enhanced, unlike in the CHOP KO case. However, the X-ray-induced stress is able to trigger innate immune signalling despite its lower efficacy in inducing damage as compared to proton irradiation, similarly to the CHOP KO. Thus, mitochondrial dysfunction also lowers the threshold at which DNA damage can induce immune signalling mechanisms. The differential response in terms of RNA and DNA binding pathways indicates that the innate immune response is independent of the RNA and DNA signalling events.

In the immune signalling enriched pathways from our analysis, several genes are characteristic of a cGAS-STING activation, including *Ifnb1*, *Il6*, *Cxcl10*, *Ccl5, Irf7*, *Ifna4*, and *Ifna5* (Supplementary Table S3). We have verified the change in mRNA level for some of these indicators of cGAS-STING activation (Supplementary Figure S3). Interestingly, analysis of the individual gene expression shows an increase in the inflammation markers in the H+C+ treated with X-ray, although the pathway does not seem to be enriched in the GO analysis. Thus, X-ray irradiation increased *Il6* levels in all the genotypes tested (H+C+, H-C+, H+C-); *Cxcl10* appeared increased in the HtrA2 and CHOP loss-of-function cells but not in the control H+C+; and *Ccl5*, *Ifnb1*, and *Ifna5* were increased in H+C+ and Htra2 loss-of-function cells but not in the CHOP loss-of-function cells.

It is interesting to note that although DNA damage-related immune responses appear for CHOP KO and HtrA2 KO cells, bystander responses are inhibited by mitochondrial dysfunction and ISR impairment.

Innate immune response has been found to be triggered by a range of events involving dysregulation of DNA homeostasis. These include microbial infection and the presence of microbial DNA in a host mammalian cell, release of mitochondrial DNA into the cytoplasm following mitochondrial damage, presence of cytoplasmic genetic material, (e.g., extranuclear chromatin, and micronuclei) due to genotoxic stress. This activates the cGAS-STING signalling mechanisms which lead through IRF3 activation to upregulation of interferon genes/innate immune enhancement [22]. The cGAS sensor of this system is located in the membrane structures; so far, the complex has been identified in the inner side of the plasma membrane, the cytoplasm, and the ER compartment as well as in the nuclear compartment. Recent studies have built a complex picture of the roles of STING beyond its cytokine, inducing functions such as autophagy, cell death, ER stress, and metabolic modulation [23]. Recent data also suggest that apart from initiating innate immunity, the cGAS-STING system in the nuclear compartment may be directly implicated in DNA repair mechanisms with a role distinct from the inflammation pathway [23]. Our data indicate that loss of HtrA2 does not increase the accumulation DNA damage induced by irradiation as determined by the MN assay; however, the accumulation of mitochondrial damage renders the mitochondria more prone to release mtDNA following an irradiation challenge. CHOP loss of function appears to increase the DNA damage accumulation following irradiation, which may relate to impairment in stress-signaling mechanisms that would support DNA repair and cellular recovery.

The studies of STING implication in autophagy have led to several working models to characterise this process. Two of them appear to involve *Atg5* and *Atg16*, which are important components of the mitophagy pathway, in addition to other types of autophagy. However, to our knowledge a mechanism for the implication of STING in mitophagy has not been proposed. On the contrary, impaired mitophagy through the PINK1/PARKIN loss of function combined with mitochondrial stress through exercise or enhanced mtDNA mutations appears to initiate an inflammatory response through the cGAS-STING pathway, thus contributing to neurodegeneration [24,25]. Mitochondrial dysfunction is thus directly linked to cGAS-STING activation, with the initiating step of this activation appearing to be the release of mitochondrial DNA and/or RNA into the cytoplasm [26].

Although evidence for the implication of inflammation in neurodegeneration is growing for a range of diseases, the specific role of cGAS-STING pathway activation is yet to be fully established. Outstanding questions remain in the cell type specific responses and their contribution to the overall neurodegeneration etiopathology and the interactions between the cGAS-STING and other signalling cascades active during neurodegeneration initiation and progression [27,28].

In the case of combined mitochondrial stress and genotoxic stress, mitochondria and the nucleus compete for metabolic resources to repair themselves [2]. Additional metabolic support through NAD+ supplementation has been demonstrated to have a positive effect in ameliorating degenerative phenotypes in a model of Alzheimer’s disease partially through a reduction in cGAS-STING activation [29].

In neurodegenerative disease, reducing cGAS-STING activation appears to have a neuroprotective role, and thus pharmacological targeting aims to decrease inflammation. In oncogenic treatments with radiotherapy, activation of this pathway has been proposed as a strategy to enhance tumour sensitivity and immune surveillance [30]. Key mechanisms for cGAS-STING activation to target tumorigenesis include facilitating the processing and presentation of tumour antigen trafficking and activation of T-cells [31]. Immunotherapy is becoming a key field in cancer therapy; thus, the profound effects of radiotherapy on the immune system cannot be ignored. As evidence indicates that cGAS-STIG activation improves the immunostimulatory effect of irradiation, the opposite effect of eliciting immunosuppression may appear through the multiple signalling pathways initiated by genotoxic stress; of particular relevance are the noncanonical NF-kB pathway and the autophagy [30]. These bidirectional actions are thus likely to interfere with clinical outcomes of combined radio-, chemo-, and immunotherapies [31].

## 3. Materials and Methods

### 3.1. Cell Culture

Four different genotypes of MEF cell lines were used for the experiments: control H+C+: HtrA2 Het (+/−), CHOP wt (+/+) wild-type; HtrA2 Het and CHOP Het do not show any functional or genetic phenotypes [14]; thus we used them as controls; H+C-: HtrA2 wt (+/+), CHOP KO (−/−); H-C+: HtrA2 KO (−/−) CHOP wt (+/+); H-C-: HtrA2 KO (−/−) CHOP KO. The cells were a gift from Dr. LM Martins [14]. The cells were grown in the DMEM media (Gibco™) supplemented with 10% fetal bovine serum and 5% Penicillin-Streptomycin (5000 U/mL) (Gibco™).

### 3.2. Genotoxic Treatment

For X-ray irradiation, a XSTRAHL XRC 160 irradiator was used (manufacturer XSTRAHL, Suwanee, GA, USA). The cells were irradiated with doses of 0.5 Gy and 2 Gy with an X-ray beam characterized by a 150 keV energy, HVL 6.57 mm Al; 20 mA; and a final debit of 0.125 Gy/min.

The cell irradiation with protons (0.5 Gy and 2 Gy with a debit of 1 Gy/min) was made using a TR-19 cyclotron. A linear energy transfer (LET) of 6.65 keV/µm corresponding to the spread-out Bragg peak (SOBP) region was used. This LET was obtained with the native 19 MeV proton beam with a filter of plexiglass inserted between the exit of the beam and the sample holder.

The treatment of cells with bleomycin (BLM) was performed by incubating them 1 h with a solution of bleomycin sulfate (SigmaAldrich, Milwaukee, WI, USA) at 5–60 μg/mL followed by 3 washes with culture medium.

### 3.3. Bystander Effect

The bystander effect was induced by using the medium transfer protocol from treated cells to nontreated cells. Immediately after the genotoxic treatments, the H+C+ control cells (directly treated, donor cells) were incubated for 24 h in fresh medium to allow the release of bystander factors. Then bystander conditioned medium was collected, filtered (0.22 μm filters, Millipore, Darmstadt, Germany) and transferred onto untreated H+C+, H+C-, H-C+ H-C- cells for 24h.

### 3.4. Cell Viability

The MTT assay was performed using the Vybrant^®^ MTT Cell Proliferation Assay Kit Thermo Fisher Scientific.

### 3.5. ATP Level Measurement

ATP levels were assessed using CellTiter-Glo (Promega) following the manufacturer’s instructions. Briefly, the cell lines were cultured in a 96-well plate, followed by drug treatment. For analysis, ATP levels were quantified using ATP standard curves using serial dilution of ATP (50 µL) solution in culture medium. The cells were incubated for 30 min. Chemiluminescence was measured using a SpectraMax (MS) spectrophotometer at a wavelength of 570 nm. Furthermore, the ATP levels were normalized to protein content, measured by the Bradford assay. The ATP data are presented relative to the H+C+ cell line.

### 3.6. Protein Level Measurement (Bradford Assay)

Protein levels were determined for normalization purposes using the Bradford Assay. Briefly, the cells cultured in 96-well plates were washed with PBS to remove residual proteins from the media and incubated with the Bradford solution (Sigma, Rödermark, Germany) for 30 min, followed by subsequent washing in PBS. In each experiment, the samples were normalized to a standard curve prepared by a BSA standard stock solution (in PBS) with serial dilutions made in the Bradford solution.

### 3.7. Gene Expression Analysis

RNA was extracted using TRIzol (Sigma) according to the manufacturer’s instructions. Reverse transcription was performed using the High-Capacity Reverse Transcription Kit (Applied Biosystems). Quantitative RT-PCR was performed with an Applied Biosystems cycler using the SYBR Green RT-PCR system. Gene-specific primers were designed and obtained from Sigma. The relative transcript levels of the target genes were normalized against *GAPDH* mRNA levels [32].

### 3.8. High Resolution Respirometry

Mitochondrial respiration was assayed at 37 °C by high-resolution respirometry using an OROBOROS Oxygraph. The DatLab software package (OROBOROS, Innsbruck, Austria) was used for data acquisition and analysis. OXPHOS Complex I- and OXPHOS Complex I- and II-driven respiratory states, reflecting the capacity for oxidative phosphorylation, were assayed in MiR05 respiration buffer (20 mM HEPES, 10 mM KH_2_PO_4_, 110 mM sucrose, 20 mM taurine, and 60 mM K-lactobionate; pH 7.1), 5 mM EGTA, 3 mM MgCl_2_, and 1 g/L BSA (fatty acid free) in the presence of saturating ADP (5–10 mM) and using the substrates malate (2 mM), glutamate (10 mM), and succinate (10 mM). Maximum electron transfer system (ETS) capacity was achieved by uncoupling the mitochondria with CCCP titrations. The difference between Complex I- and II-driven ETS and OXPHOS provides the ETS excess capacity, which is available to drive processes other than oxidative phosphorylation without competing with ATP production.

### 3.9. Micronuclei (MN) Analysis

The cells were cultured on glass coverslips in 24-well plates. For the study of directly irradiated cells, cytochalasin B was added at 3 μg/mL 4 h following irradiation and then incubated for 20 h. The cells were then washed once with PBS and fixed with methanol– acetic acid (9:1) and stored at 4 °C for 2 h. The Acridine Orange at 10 μg/mL was added for 10 min followed by washing in PBS and mounting. Then the MN were observed by fluorescence microscopy. Micronuclei were scored according to the Fenech criteria in 1000 binucleated cells [33]. In the case of bystander studies, H+C+, H+C-, H-C+, and H-C- cells were first incubated for 24 h with conditioned medium of H+C+ control treated cells, and then the bystander cells were treated with cytochalasin B similarly to the directly treated cells.

### 3.10. Statistical Analyses

The data are presented as mean values, and error bars indicate ±SD or ±SEM as noted. Inferential statistical analysis was performed using the Prism and StatMate software packages (http://www.graphpad.com). The significance level is indicated as **** *p* < 0.0001, *** *p* < 0.001, ** *p* < 0.01, and * *p* < 0.05, and NS indicates *p* > 0.05.

### 3.11. RNA Sequencing Analysis

RNA sequencing has been outsourced to NOVOGENE.

Briefly, the RNA sequencing and analysis comprised the following steps: total RNA quantification and quality control, mRNA enrichment, double stranded cDNA synthesis, end-repair, poly-A and adaptor addition, fragments selection and PCR, Library quality assessment, and Illumina sequencing. A total amount of 1 µg RNA per sample was used as input material for the RNA sample preparations. The sequencing libraries were generated using NEBNext^®^ Ultra^TM^ RNA Library Prep Kit for Illumina^®^ (NEB, Ipswich, MA, USA) following the manufacturer’s recommendations, and index codes were added to attribute sequences to each sample. The clustering of the index-coded samples was performed on a cBot Cluster Generation System using PE Cluster Kit cBot-HS (Illumina) according to the manufacturer’s instructions. After cluster generation, the library preparations were sequenced on an Illumina platform, and paired-end reads were generated. Raw data (raw reads) of FASTQ format were firstly processed through fastp. In this step, clean data (clean reads) were obtained by removing reads containing adapter and poly-N sequences and reads with low quality from raw data. All the downstream analyses were based on the clean data with high quality.

Reference genome and gene model annotation files were downloaded from the genome website browser (NCBI/UCSC/Ensembl) directly. Paired-end clean reads were mapped to the reference genome using HISAT2 software. Because transcriptome annotations are still incomplete, most RNA-seq studies will reveal novel genes and transcripts. The Stringtie was used to assemble the set of transcript isoforms of each bam file obtained in the mapping step. gffcompare can compare Strintie assemblies to reference annotation files and help sort out new genes from known ones. Feature counts was used to count the read numbers mapped of each gene, including known and novel genes. Then the RPKM of each gene was calculated on the basis of the length of the gene and thereads count mapped to this gene. RPKM, Reads Per Kilobase of exon model per Million mapped reads, considers the effect of sequencing depth and gene length for the reads count at the same time and is currently the most commonly used method for estimating gene expression levels.

### 3.12. Differential Expression Analysis and Pathway Enrichment Analysis

Differential expression analysis was performed using the webtool iDEP (integrated Differential Expression and Pathway analysis) developed by Ge, S.X., and colleagues (2018) [34]. Read count data (Supplementary Table S1) was uploaded in the form of a .csv file in iDEP 0.96 (http://bioinformatics.sdstate.edu/idep96/, accessed on 6 November 2022). In the preprocessing step, the default filter 0.5 CPM was used to filter out lowly expressed genes, meaning that genes which expressed fewer than 10 reads in all samples were removed. EdgeR log2(CPM+c) was selected to reduce the variability and normalize the read counts data. The DESeq2 package embedded in iDEP was employed to identify the differentially expressed genes. An analysis of DEGs was performed between the mock and irradiated samples for selected genotypes with the DESeq2 method by using a threshold of false discovery rate (FDR) of 0.1 and a fold change of minimum 2 [34].

Gene set enrichment analysis was performed on iDEP to determine the shared biological functions of differentially expressed genes on the basis of significant GeneOntology (GO) terms. GO terms including biological process, molecular function, and cellular component and only the categories that provided significant enrichment were reported.

## 4. Conclusions

**Figure 9:**
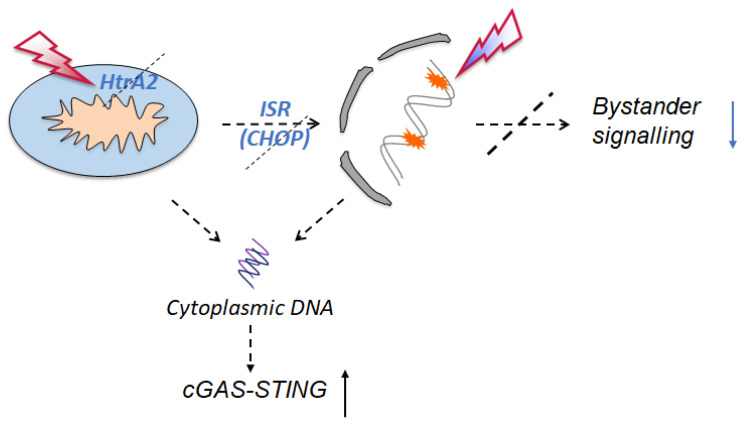
Mitochondrial stress and DNA damage following genotoxic stress result in release of genetic material into the cytoplasm. This induces the activation of innate immunity via the cGAS-STING pathway. Loss of mitochondrial quality control through HtrA2 loss of function, and ISR signalling integrity through CHOP loss of function, reduce the threshold for the cGAS-STING activation, which may affect cellular homeostasis differently in different disease conditions.

Mitochondria dysfunction and DNA damage initiate stress signalling mechanisms that expand beyond these two organelles and involve multiple intracellular and intercellular communication pathways. Here we demonstrate that impaired mitochondrial quality control and ISR apparatus lower the threshold for genotoxicity to induce innate immune pathways through cGAS-STING. These are also able to impair the intercellular transmission of DNA damage. cGAS-STING activation may have different consequences in different disease contexts; thus, targeted approaches for modulating immune responses need to be considered (Figure 9).

## Data Availability

The data are included in the paper and the Supplementary Materials.

## Supplementary Materials

The following supporting information can be downloaded at: https://www.mdpi.com/article/10.3390/ijms24065891/s1.
