# Supplementary material for: Impaired Integrated Stress Response and Mitochondrial Integrity Modulate Genotoxic Stress Impact and Lower the Threshold for Immune Signalling
Source: Int J Mol Sci. 2023 Mar 20;24(6):5891. doi: 10.3390/ijms24065891 (PMC10059776; doi:10.3390/ijms24065891)
Supplement: Supplementary file 1 [file ijms-24-05891-s001.zip › Supplementary Material DIS and NM-v3.pdf]

Impaired integrated stress response and mitochondrial integrity modulate genotoxic stress impact and lower the threshold for immune signalling.

Mihaela Temelie[1], Rubab Talpur[2], Marta Dominguez-Prieto[2], Ayanda Dantas Silva[2], Constantin Cenusa[3], Liviu Craciun[4], Diana Iulia Savu[1,*], Nicoleta Moisoi[2,*]

SUPPLEMENTARY MATERIAL

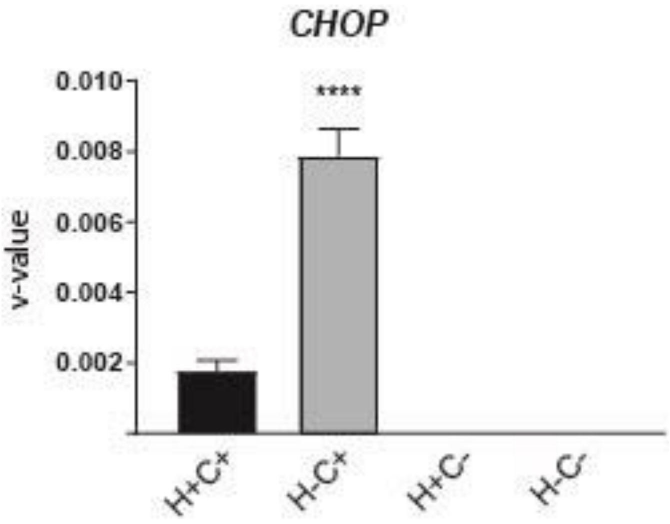

Supplementary Figure S1. Loss of HtrA2 enhances the transcriptional level of CHOP. The data is presented as mean ± SEM. The statistical significance was analysed with One-way ANOVA with multiple comparison's and the difference versus the H+C+ genotype is indicated in the figure (**** $p < 0.0001$).

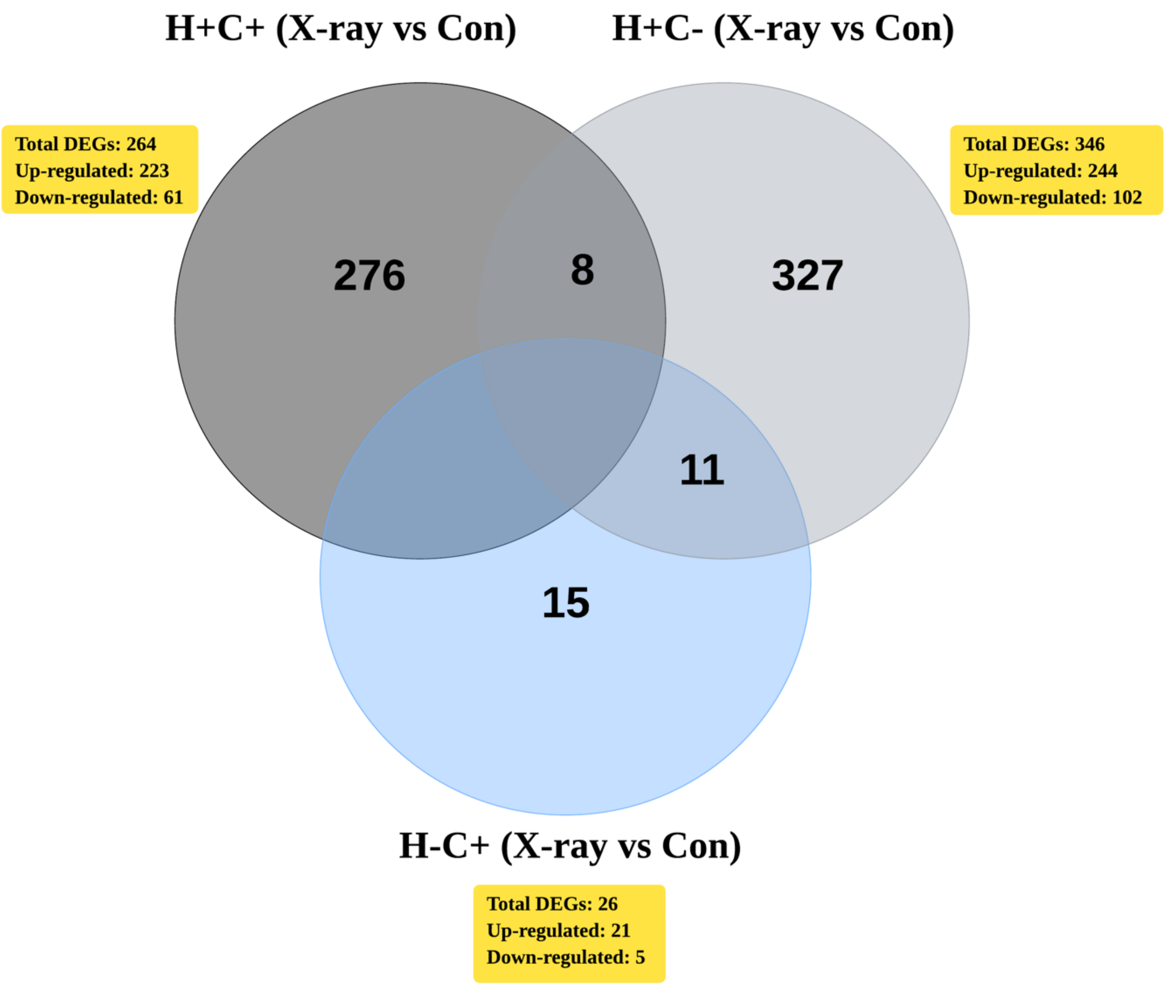

Supplementary Figure S2. Venn diagram representing common and unique differentially expressed genes (DEGs) in the three comparisons identified by RNA-Seq. Boxes in yellow showing the number of upregulated and down-regulated genes. Figure shows the numbers DEGs for each comparison and the overlap between X-Ray induced genes in H+C+, H+C- and H-C+ genotypes.

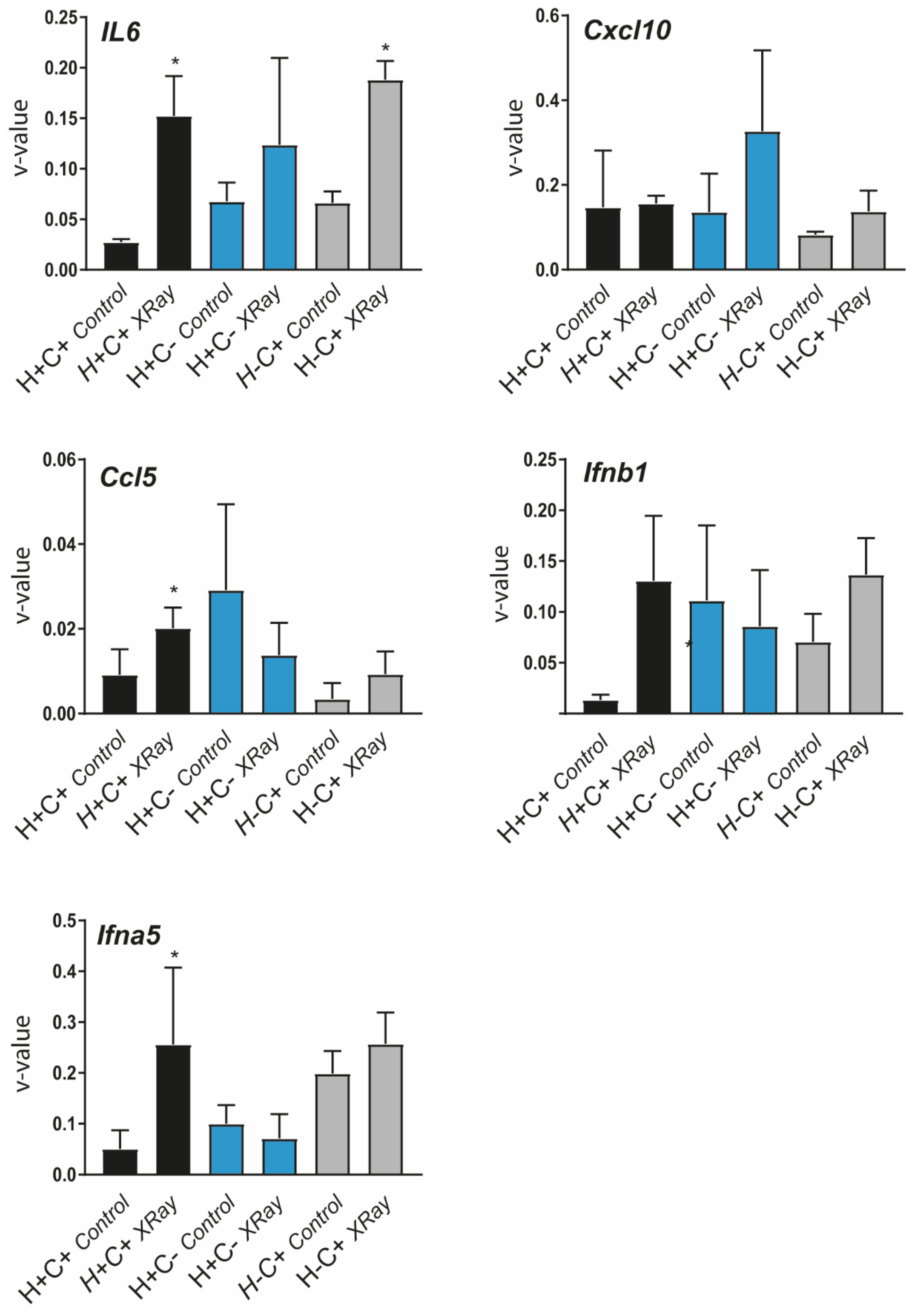

Supplementary Figure S3. Transcriptional changes of inflammation markers linked with the cGAS- STING activation. The data is presented as mean ± SD from n=2-4 replicates. The statistical significance was analysed with Student t-test between treatment and control for each genotype. * indicates p < 0.05.

Supplementary Table S1 (excel file). Number of reads identified for each gene for the individual samples sequenced. The sample IDs use ‘1’ and ‘0’ for the ‘+’ and ‘-‘ genotypes’ identification as requested for analysis purposes by Novogene. C and D followed by a number are indicating control samples (mock) for X-Ray and Proton respectively, while X and P followed by number are indicating samples treated with X-Ray or Proton irradiation respectively. Gene IDs are provided.

Supplementary Table S2. Number of genes upregulated and downregulated in the selected treatments.

genotype	X Ray-up	X Ray-down	Proton-up	Proton-down
H+C+	223	61	263	35
H+C-	244	102	-	-
H-C+	21	5	-	-

Supplementary Table S3 (excel file). Enriched GO Biological Process and GO Molecular Component Pathways. The genes identified in the upregulated and downregulated enriched pathways are listed for each comparison. The samples IDs used in the comparisons are indicated. The sample IDs use ‘1’ and ‘0’ for the ‘+’ and ‘-‘ genotypes’ identification as requested for analysis purposes by Novogene. C and D followed by a number are indicating control samples (mock) for X-Ray and Proton respectively, while X and P followed by number are indicating samples treated with X-Ray or Proton irradiation respectively.
